# Frequencies of CYP2B6^∗^4,^∗^5, and ^∗^6 Alleles within an Iranian Population (Mazandaran)

**DOI:** 10.1155/2021/8703812

**Published:** 2021-12-02

**Authors:** Mohammad Bagher Hashemi-Soteh, Elaheh Hosseini, Shokoufeh Fazelnia, Faramarz Ghasemian-Sorbeni, Sara Madahian, Mohammad Reza Shiran

**Affiliations:** ^1^Immunogenetic Research Center, Molecular and Cell Biology Research Center, Faculty of Medicine, Mazandaran University of Medical Sciences, Sari, Iran; ^2^Novin Genetic Diagnostic Laboratory, FarahAbad Boulevard, Sari, Mazandaran, Iran; ^3^The Health of Plant and Livestock Products Research Center, Mazandaran University of Medical Sciences, Sari, Iran

## Abstract

**Background:**

The human CYP2B subfamily consists of one functional gene (CYP2B6) and one pseudogene (CYP2B7P). Cytochrome P450 2B6 (CYP2B6) is a highly polymorphic enzyme that shows marked interindividual and interethnic variations. Currently, 38 alleles have been described, and some of the allelic variants have been associated with low enzyme activity. The aim of this study was to investigate the frequencies of CYP2B6^∗^4, CYP2B6^∗^5, and CYP2B6^∗^6 alleles in the Mazani ethnic group among Iranian Population.

**Methods:**

The study was conducted in 289 unrelated healthy volunteers. DNA was extracted from peripheral blood and analyzed by the PCR-RFLP protocol. The PCR product was digested with restriction enzymes and then separated using agarose gel electrophoresis.

**Results:**

The frequency of CYP2B6^∗^4, CYP2B6^∗^5, and CYP2B6^∗^6 in this study was 34.60%, 7.26%, and 34.54%, respectively.

**Conclusion:**

The frequency of the CYP2B6^∗^4 allele in the Mazani ethnic group was much higher (34.60%) than other population. The frequency of CYP2B6^∗^6 (34.54%) also was higher than its frequency in other previously reported population. But the frequency of CYP2B6^∗^5 in this study was lower than expected. These results will be useful in understanding the ethnic diversity in Iranian population and offer a preliminary basis for more rational use of drugs that are substrates for CYP2B6 in this population.

## 1. Introduction 

Polymorphisms are the cause of 15–30% of individual difference in the drug metabolism [1, 2]. The human CYP is a supergene family which is expressed in the liver. 57 polymorphic genes containing a large number of SNVs and CNVs belong to this supergene family [3]. One of the most polymorphic gene in this family is CYP2B6, which is located on 19q13.2 within CYP2 gene cluster [4, 5].

Cytochrome p402B6 (CYP2B6) is known as one of the important subclasses for drug metabolizing enzyme in the liver and other organs. Polymorphisms of this gene cause differences in transcriptional regulation, splicing, and expression of mRNA and protein [5].

CYP2B6 is involved in the metabolism and metabolic activation of many clinically important drugs such as antiretrovirals, efavirenz, and nevirapine; the antidepressants bupropion, sertraline; the antiestrogen tamoxifen; the synthetic opioid methadone; the anti-Parkinsonian selegiline; the antimalarial artemisinin, ketamine, and propofol; and cytotoxic prodrugs cyclophosphamide, ifosfamide, thiotepa, and procarbazine [6–9].

The CYP2B6 gene is mainly expressed in the liver cells, where it makes about 3–5% of the total microsomal P450 pool [10–12]. It is also active at lower levels in extrahepatic tissues, including the intestine, kidney, lung, skin, and brain [13, 14]. CYP2B6 expression levels in human livers vary from 20 to 250 folds between different individuals, while CYP2B6 activity in liver microsomes varies more than 100 folds [15–17]. Transcriptional regulation is considered to be one of the major contributors to this variability. CYP2B6 is highly inducible by phenobarbital-type compounds as well as many other typical inducers of CYP3A4 in a dose-dependent manner [18–20]. Furthermore, the differences in gene regulation and genetic polymorphisms largely contribute to interindividual variability in CYP2B6 activity. Currently, 38 alleles have been described for CYP2B6 [21]. Low enzyme activity is the result of some allelic variants. These variants include single nucleotide polymorphisms (SNPs) located in the coding region, such as CYP2B6^∗^4A (c.785 A > G), CYP2B6^∗^5A (c.1459 C > T), and CYP2B6^∗^6A (c.516 G > T). Among these alleles, CYP2B6^∗^6 as an allele with high frequency in different ethnic and population (15–60%) is noticeable [5]. In the present study, we examined the frequencies of CYP2B6^∗^4 (rs2279343), CYP2B6^∗^5 (rs3211371), and ^∗^6 (rs3745274) mutant alleles in the Mazani ethnic group among Iranian population.

## 2. Materials and Methods

### 2.1. Subjects

289 unrelated healthy volunteers of Mazani origin, residing in Mazandaran, a northern province in Iran, were enrolled in the study. The investigation workflow was approved by the Research Ethics Committee of Mazandaran University of Medical Sciences. All subjects were included in the study after signing the consent form.

### 2.2. Genomic DNA Extraction

5–10 ml venous blood was obtained from each subject and stored in an Na-EDTA tube at −25°C until processing. Lymphocytic genomic DNA was extracted by the Nucleon BACCII method [22], followed by DNA concentrations measurement using the NanoDrop instrument (Biowave, UK).

### 2.3. PCR Amplification of the CYP2B6 Alleles

Allele-specific PCR was carried out to detect CYP2B6^∗^4, CYP2B6^∗^5, and CYP2B6^∗^6 alleles and their genotype frequency, respectively. The specific primers were used to amplify each CYP2B6 allele separately (Table 1). The total volume of each PCR reaction was 25 *μ*l containing 0.6 *μ*l forward primers and 0.6 *μ*l reverse primers, 2 *μ*l DNA template, and 11 *μ*l EmeraldAmp PCR master mix (Takara Bio Inc., Japan), up to 25 *μ*l dH_2_O. The PCR reactions were carried out with the following conditions: 93°C, 40 s; annealing temperature for 40 s; 72°C, 40 s; for 35 cycles. PCR products were visualized on 1% agarose gel.

### 2.4. Genotyping of the CYP2B6^∗^4 Allele

**Figure 1:**
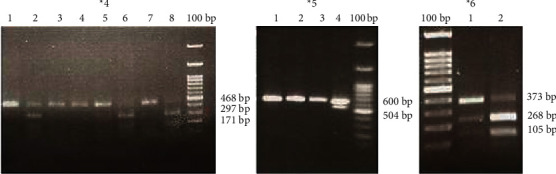
The restriction analysis result for CYP2B6^∗^4, ^∗^5, and ^∗^6 variants in Mazani ethnic group people. StyI enzyme cuts the normal variant to 56, 116, 171, and 297 bp and mutated allele of ^∗^4 to 56, 116, and 468 bp. BglII does not cut the normal variant (600 bp) and just make the mutant ^∗^5 allele to 96 and 504 bp. Finally, BsrI digest the normal variant to 28, 105, 268 bp and ^∗^6 allele to 28 and 373 bp.

PCR products of CYP2B6^∗^4 revealed a 640 bp band and were digested using StyI restriction enzyme as previously reported [23, 24]. 0.3 *μ*l of StyI enzyme and 1 *μ*l enzyme buffer were added to 6 *μ*l of CYP2B6^∗^4 PCR product and 3 *μ*l distilled water. The reaction tubes were incubated overnight at 37°C prior to analysis on 3% agarose gel. Mutant allele created three different bands (56, 116, and 468 bp), while the normal case showed four separate bands, containing 56, 116,171, and 297 bp. The size of the DNA fragments was determined by comparing with a standard size marker DNA ladder (Figure 1).

### 2.5. Genotyping of the CYP2B6^∗^5 Allele

The PCR product for CYP2B6^∗^5 revealed a 600 bp band. After digestion using the BglII restriction enzyme, mutant allele showed two bands, 504 bp and 96 bp, but the enzyme did not cut the wild type 600 bp original band, genotype ^∗^1/^∗^1. The reaction tubes were incubated overnight at 37°C prior to analysis on a 3% agarose gel (Figure 1).

### 2.6. Genotyping of the CYP2B6^∗^6 Allele

The PCR product for CYP2B6^∗^6 was a 401 bp fragment. After digestion using the BSrI restriction enzyme, three bands were created in the gel including 28, 105, and 268 bp for the wild type. Also, the enzyme on the mutant allele produced two distinct bands including 28 bp and 373 bp (Figure 1).

### 2.7. DNA Sequencing

**Figure 2:**
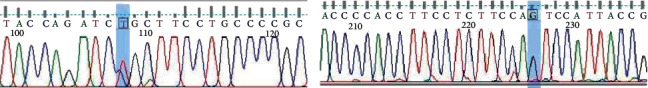
DNA sequence chromatogram showing nucleotide change position. (a) A missense nucleotide transition C > T in c.1483 of CYP2B6 gene, a heterozygous sample for CYP2B6^∗^5 (rs 3211371) polymorphism. (b) A normal sample with major allele G in c.540 G > T of CYP2B6 gene for CYP2B6^∗^6 (rs 3745374) polymorphism.

In order to confirm the RFLP results, some samples were subjected to DNA sequencing using specific primers (Table 2). A DNA sequence analysis software, GeneRunner (https://www.generunner.com), was applied along with using reference sequences from GenBank database. Finch TV, a DNA sequence chromatogram viewer software (Geospiza, Inc., USA), also was applied (Figure 2) to view nucleotide changes. Figure 2 shows two nucleotide change, CYP2B6^∗^5 (rs 3211371) and CYP2B6^∗^6 (rs 3745374), in CYP2B6 gene [25, 26].

## 3. Results

In total, 289 individuals from Mazandaran province (Mazani ethnics) were tested for 3 different polymorphisms in Cyp2B6 gene. Frequencies of the three polymorphisms including CYP2B6^∗^4, CYP2B6^∗^5, CYP2B6^∗^6 in 289 individuals are provided in Table 3. The frequency of polymorphic CYP2B6 alleles responsible for impaired drug metabolisms CYP2B6^∗^4, ^∗^5, and ^∗^6 was 34.60%, 7.26%, and 34.54%, respectively (Table 3).

## 4. Discussion

Different ethnic groups live in various parts of Iran. These ethnic groups include Persian, Azari, Turkmen, Kurd, Arab, Lor, Balouch, Gilaki, and Mazani [27]. Whereas CYP2B6 genetic polymorphisms have previously been assessed in other population and southern Iranians [21], there is a lack of data in the Mazani ethnic group.

The CYP2B6 polymorphism is characterized by numerous variants in both coding and noncoding regions of the gene. The website of CYP alleles (https://www.pharmvar.org) lists 38 distinct alleles for CYP2B6 gene (accessed April 2021). In human livers, CYP2B6^∗^6 has been associated with lower protein expression and lower hydroxylation activity towards efavirenz and bupropion [28]. CYP2B6^∗^6 variant 516G > T (Q172H) is involved in the posttranscriptional mechanism and causes an aberrant splicing which results in missing of exons 4–6 in mRNA transcripts and causes lower expression of CYP2B6 protein [29]. In vivo, CYP2B6^∗^6 has been consistently associated with higher plasma levels of efavirenz during treatment [30]. At least half of the patients who receive efavirenz faced with central nervous system (CNS) side effects are thought to be a reflect of higher efavirenz plasma concentrations [31, 32]. Interestingly, Gatanaga et al. were able to successfully employ CYP2B6^∗^6 genotyping to reduce the therapeutic dose of efavirenz and improve the CNS-related side effects [33]. The CYP2B6^∗^6 variant allele has a frequency between 15% and over 50% across different populations, which has the highest frequencies in African and the lowest in Asians populations, respectively (Table 4). Ethnicity is an important variable contributing to interindividual variability in the drug metabolism, response, and toxicity [34]. The 34.54% frequencies of the CYP2B6^∗^6 allele found in the Mazani ethnic group was considerably higher than those found in Caucasian, African-American, Chinese, Japanese, and Korean populations with average frequency of 12–35% and is comparable to those reported in Africans (Table 3).

Also, in human livers, CYP2B6^∗^5 is associated with lower protein expression, bupropion hydroxylation, and S-mephenytoin N-demethylation [12]. This allele shows the highest (12.8%) and the lowest (0.1%) frequency in Europe and East Asia, respectively [35]. Despite lack of CYP2B6^∗^5 alleles in Korean or Chinese populations, its frequency in different Europian countries is considerable and around 10–15% (Table 4). The 7.26% frequency of CYP2B6^∗^5 found in the Mazani ethnic group in this study is comparable to those found in African and Japanese. By contrast, it occurs at a relatively lower frequency in Caucasian and African-American (Table 4). Notably, no clear effect on CYP2B6 functionality has been revealed for CYP2B6^∗^5 [35]. Although in vitro studies have clearly represented an association between CYP2B6^∗^5 variant and decreased activity and protein expression [36], but in vivo studies have not shown any effect of CYP2B6^∗^5 on efavirenz pharmacokinetics and reported lack of a significant phenotype-genotype association [37, 38]. This difference in results can be explained by an increased specific activity of the gene product towards efavirenz, which may compensate an inherent low expression [39, 40]. Thus, it is important for future studies to investigate under which conditions a lower frequency of CYP2B6^∗^5 could be clinically important.

Interestingly, CYP2B6^∗^4, emerged by a gain of function mutation, is relevant to high level of gene expression and may lead to a moderate substrate-dependent effects. As a result, a disruption occurs in the hydroxylation process in the metabolism of some relevant drugs such as bupropion, efavirenz, propofol, and clotiazepam [5]. A relatively low prevalence for ^∗^4 allele in different populations was demonstrated by previous investigations. This allele frequency was reported 5% in Germany [41], 2.2% in Caucasian in New Zealand, 3.3% in Chinese, and 6% in United States, respectively [42–45]. The results of current research showed a frequency of 34.60% for the CYP2B6^∗^4 minor allele (G) (Table 3), which is significantly higher than its frequency in other parts of the world. Table 4 provides the frequency of some other relevant studies from different countries.

The global distribution for CYP2B6^∗^6 is reported 73% and 26% for the G and T alleles, respectively [51]. The frequency of CYP2B6 minor allele (T) is estimated about 21.5% in East Asian and 38.1% in South Asian [52] (Table 4). In Pakistan population, eastern neighbor of Iran, the frequency of CYP2B6^∗^6 minor allele (T) is reported about 33.8% [51]. Frequency of CYP2B6^∗^6 achieved in the current study is 34.54% (Table 3), slightly more than East Asia and Pakistan. According to the 1000 Genome project, the lowest frequency of CYP2B6^∗^4 minor allele (G) is reported from European with 8.8% and in South Asian with highest frequency of 25.2%, respectively (Table 5) [52]. Frequency of CYP2B6^∗^4 achieved in the current study is 34.60% (Table 3).

## 5. Conclusion

The result of this study will aid in understanding the ethnic diversity of the Iranian population and offer a preliminary basis for more rational use of drugs that are substrates for CYP2B6 in this population.

## Figures and Tables

**Table 1:** Specific primers for amplification and evaluation of each CYP2B6 defective allele.

CYP2B6 allele	Specific primer pairs	Annealing Tm	PCR product sizes
^∗^4	F: 5′GACAGAAGGATGAGGGAGGAA3′	59°C	640 bp
	R: 5′CTCCCTCTGTCTTTCATTCTGT3′		

^∗^5	F: 5′ACAAGAATCATTTGAACCACCTG3′	59°C	600 bp
	R: 5′AGTCAGAGCCATTGTCTACAG3′		

^∗^6	F: 5′TCTCGGTCTGCCCATCTATAAACT3′	59°C	401 bp
	R: 5′CCTGACCTGGCCGAATACA3′		

**Table 2:** Specific primers for PCR sequencing.

CYP2B6 allele	Specific primer pairs	Annealing Tm	PCR product sizes
^∗^5	F: 5′AGCGGATTTGTCTTGGTGAA 3′	59°C	225 bp
	R: 5′ACACTGAATGACCCTGGAATCC 3′		

^∗^6	F: 5′AGCCTCTCGGTCTGCCCATCTATA3′	64°C	423 bp
	R: 5′CCTGTCCCTCTCCGTCTCCCTGA′		

**Table 3:** The allele and genotype frequencies of CYP2B6^∗^4, ^∗^5, and ^∗^6 in Mazani ethnic people (*n* = 289).

Variant	Allele frequency (%)	Genotype frequency (%)
rs2279343-^∗^4	34.60	AA: 136 (47.05), AG: 106 (36.67), GG: 47 (16.26)
rs3211371-^∗^5	7.26	CC: 249 (86.15), CT: 38 (13.14), TT: 2 (0.69)
rs3745274-^∗^6	34.54	GG: 138 (47.75), GT: 103 (35.64), TT: 48 (16.60)

**Table 4:** The frequencies of CYP2B6 different alleles in different populations.

Population	*N*	CYP2B6^∗^4 (%)	CYP2B6^∗^5 (%)	CYP2B6^∗^6 (%)	Reference
Southern Iranian	206	10.4	2.4	23.1	[21]
African	166	—	2	42	[45]
Japanese	265	9.3	1.1	—	[46]
Chinese	139	3	—	25.8	[47]
Korean	88	4.5	—	15.9	[48]
United Kingdom	135	2.2	12.2	28.1	[49]
Italian	174	1.82	17.3	29.1	[50]
German	121	5	9.5	25	[41]
American	60	6	3	28	[45]
African-American	93	2	5	34	[45]
Caucasian	215	4	10.9	25.6	[12]
Current study	100	43	0.08	48	—

**Table 5:** CYP2B6^∗^6 and ^∗^4 allele frequencies as reported in various superpopulations in the 1000 Genome project.

Population	CYP2B6^∗^6 (rs3745274)		CYP2B6^∗^4 (rs2279343)	
	G (%)	T (%)	A (%)	G (%)
African	62.6	37.4	82.9	12.9
American	62.7	37.3	83.4	16.6
East Asian	78.5	21.5	85.3	14.7
European	76.4	23.6	91.2	8.8
South Asian	61.9	38.1	74.8	25.2

## Data Availability

The data used to support the findings of this study are included within the article and are made available from the corresponding author upon request.
